# Measuring executive function in control subjects and TBI patients with question completion time (QCT)

**DOI:** 10.3389/fnhum.2015.00288

**Published:** 2015-05-19

**Authors:** David L. Woods, E. William Yund, John M. Wyma, Ron Ruff, Timothy J. Herron

**Affiliations:** ^1^Human Cognitive Neurophysiology Laboratory, VANCHCSMartinez, CA, USA; ^2^Department of Neurology, University of California, DavisSacramento, CA, USA; ^3^Center for Neurosciences, University of California, DavisDavis, CA, USA; ^4^UC Davis Center for Mind and BrainDavis, CA, USA; ^5^Department of Psychiatry, University of California, San FranciscoSan Francisco, CA, USA

**Keywords:** aging, gender, reaction time, executive function, head injury, reading

## Abstract

Questionnaire completion is a complex task that places demands on cognitive functions subserving reading, introspective memory, decision-making, and motor control. Although computerized questionnaires and surveys are used with increasing frequency in clinical practice, few studies have examined question completion time (QCT), the time required to complete each question. Here, we analyzed QCTs in 172 control subjects and 31 patients with traumatic brain injury (TBI) who completed two computerized questionnaires, the 17-question Post-Traumatic Stress Disorder (PTSD) Checklist (PCL) and the 25-question Cognitive Failures Questionnaire (CFQ). In control subjects, robust correlations were found between self-paced QCTs on the PCL and CFQ (*r* = 0.82). QCTs on individual questions correlated strongly with the number of words in the question, indicating the critical role of reading speed. QCTs increased significantly with age, and were reduced in females and in subjects with increased education and computer experience. QCT *z*-scores, corrected for age, education, computer use, and sex, correlated more strongly with each other than with the results of other cognitive tests. Patients with a history of severe TBI showed significantly delayed QCTs, but QCTs fell within the normal range in patients with a history of mild TBI. When questionnaires are used to gather relevant patient information, simultaneous QCT measures provide reliable and clinically sensitive measures of processing speed and executive function.

## Introduction

Questionnaire completion engages a set of complex cognitive processes. Prior to question delivery, and between consecutive questions, subjects must maintain overall task engagement and alertness (Stuss, 2011) while simultaneously focusing attention and avoiding distractions (Commodari and Guarnera, 2008). Subjects must read and analyze each question, placing demands on long-term and working memory (Christopher et al., 2012). Subjects must then evaluate the responses, select the most appropriate option, and respond accordingly. All of these processes depend on executive functions and show age-related declines (Brand and Markowitsch, 2010). Not surprisingly, the overall completion time for questionnaires increases with age (Malhotra, 2008) in a manner that parallels the age-related slowing seen on neuropsychological tests of executive function and processing speed (Salthouse, 2000; Woods et al., 2015a). Here, we examine the use of individual question completion times (QCTs) as a measure of cognitive function.

Questionnaires are widely used in clinical research to gather information about disease symptoms and severity and to evaluate treatment outcomes. For example, patients with traumatic brain injury (TBI) may be asked to complete a variety of questionnaires investigating symptom severity (Sullivan and Garden, 2011; McLeod and Leach, 2012; Soble et al., 2014), quality of life (von Steinbüchel et al., 2010), depression (Richter et al., 1998), alcohol use (Conigrave et al., 1995), sleep disorders (Verma et al., 2007), Post-Traumatic Stress Disorder (PTSD; Blanchard et al., 1996), and cognitive problems experienced in everyday life (Broadbent et al., 1982). These questionnaires are typically administered in paper and pencil format, and, in most cases, questionnaire completion is self-paced and completion time is not recorded. There has been an increase in recent years of questionnaires on PCs and tablet devices that facilitate data management and ease of administration. In addition, computer-based questionnaires permit the precise recording of QCTs to individual questions. In the current study, we evaluated the utility of QCTs as a measure of cognitive function using computer-based delivery of the questions on the PTSD Checklist (PCL; Weathers et al., 1993; Blanchard et al., 1996) and the Cognitive Failures Questionnaire (CFQ; Broadbent et al., 1982).

By analyzing the completion time of individual questions, we were able to analyze the relationship between QCTs and rating scores on individual items, and between median QCTs and overall questionnaire scores. This permitted us to address two questions: (1) Would subjects take longer to respond to questions on which they gave higher disability ratings? and (2) Would subjects with greater self-reported disability show elevated QCTs? Analyzing QCTs to individual questions also permitted an analysis of the relationship between question length, grammatical complexity, and completion time. We anticipated that subjects would require more time to respond to longer and more complex questions.

In addition to examining the factors contributing to QCTs on individual questions, we analyzed the relationship between QCTs, age, and education, factors that correlate with overall questionnaire completion time (Allenby et al., 2002) as well as with performance on neuropsychological tests of processing speed (Woods et al., 2015b) and executive function (Woods et al., 2015a). In addition, since female subjects typically show superior levels of verbal ability and reading achievement (Lynn and Mikk, 2009), we also analyzed the influence of sex. Finally, we analyzed the influence of computer use, since subjects who used computers would have more experience with processing and responding to questions delivered by computer.

We also hypothesized that QCT measures would correlate with the results of other neuropsychological tests, including measures of crystalized intelligence such as the Wechsler Test of Adult Reading (WTAR) (2001), tests of processing speed such as choice reaction time (CRT; Woods et al., 2015b), and tests of executive function such as the Trail Making Test, part B (Tombaugh, 2004; Woods et al., 2015a). However, insofar as the cognitive demands of questionnaire completion were similar across questionnaires, we expected higher correlations between QCTs on the two questionnaires than between QCTs and the results of the aforementioned cognitive tests.

Finally, we also analyzed the clinical sensitivity of QCT measures in a group of patients with traumatic brain injury (TBI) who completed both questionnaires. The TBI group included 27 patients who had suffered mild TBI (mTBI), and four patients who had been hospitalized following severe TBI (sTBI). As a group, the TBI patients had shown small but significant deficits on some measures of processing speed (Hubel et al., 2013), working memory (Woods et al., under review), and executive function (Woods et al., 2015a, under review), with more severe deficits observed in sTBI than mTBI patients.

## Methods

### Participants

We studied 172 control subjects and 31 TBI patients, whose demographic characteristics are summarized in Table 1. The control subjects ranged in age from 18 to 82 years (mean age = 39.9 years) and had an average of 14.8 years of education. Fifty-nine percent of the control subjects were male (Table 1). Most control subjects were recruited from advertisements on Craigslist^1^, while the remainder was recruited from pre-existing control-subject populations. Control subjects were required to meet the following inclusion criteria: (a) fluency in the English language; (b) no current or prior history of psychiatric illness; (c) no current substance abuse; (d) no concurrent history of neurologic disease known to affect cognitive functioning; (e) on a stable dosage of any required medication; (f) auditory functioning sufficient to understanding normal conversational speech; and (g) visual acuity normal or corrected to 20/40 or better. Subject ethnicities were 64% Caucasian, 12% African American, 14% Asian, 10% Hispanic/Latino, 2% Hawaiian/Pacific Islander, 2% American Indian/Alaskan Native, and 4% “other.”

**Table 1:** **Summary of demographic characteristics and questionnaire performance metrics from control subjects and patients with TBI**.

	N	AGE	EDU	C-use	PCL	CFQ	PCL-QCT	CFQ-QCT	PCL QCT-z	CFQ QCT-z
CONTROL	172	39.9	14.8	5.24	33.0	48.6	6.83	6.37	0.00	0.00
TBI (all)	31	36.3	13.6	4.81	53.3	74.4	6.93	7.34	−0.14	0.33
mTBI	27	35.0	13.7	4.72	54.6	76.9	6.67	7.06	−0.27	0.21
sTBI	4	44.8	13.0	5.51	44.5	57.3	8.66	9.23	0.83	1.29

*AGE: mean age. EDU: years of education. C-use: mean computer use in hours/day. PCL: Post-traumatic stress disorder checklist total score. CFQ: Cognitive failures questionnaire total score. QCT: Question completion times (QCTs), in seconds. Z-scores were derived from QCTs using regression functions that factored out the influences of age, education, sex, and computer use in the control population. The results are shown for all TBI patients and for subgroups of patients with mild and severe TBI (mTBI and sTBI)*.

The TBI patients were recruited from the local military Veteran patient population in the Veterans Affairs Northern California Health Care System (VANCHCS). All patients had received complete clinical workups and diagnosis and were tested more than 1 year after their TBI incident. The patients included 30 males and one female, all between the ages of 20 and 61 years (mean age = 36.3 years), with an average of 13.6 years of formal education (see Table 1). Twenty seven of the patients had suffered mTBI, with one or more combat-related incidents resulting in a cumulative loss of consciousness of less than 30 minutes, no hospitalization, and less than 24 hours of post-traumatic amnesia. Four patients had suffered sTBI with hospitalization, coma duration exceeding 8 hours, and post-traumatic amnesia exceeding 72 hours. Patient ethnicities were 71% Caucasian, 9.7% African-American, 9.7% Hispanic, 6.5% Pacific Islander, and 3.2% Asian. Additional details about the patients can be found in a companion publication (Woods et al., 2015a).

All patients and control subjects signed written consent forms approved by the institutional review board (IRB) of the Veterans Affairs Northern California Health Care System (VANCHCS) and were paid for their participation. All subjects had completed a demographic questionnaire that included questions regarding the number of years of formal education and the number of hours per day that they used computers.

### Apparatus and Stimuli

The PCL and CFQ are typically self-administered, pen-and-paper tests that take 2–5 minutes to complete. The PCL is used to assess PTSD symptoms and assist in PTSD diagnosis and treatment evaluation. Each question presents a PTSD symptom and asks the subject to rate symptom severity over the past month. Subjects complete 17 questions, each based on the diagnostic criteria of the DSM-IV (Foa and Tolin, 2000), with a 5-point Likert scale rating ranging from 1 (“Not at all”) to 5 (“Extremely”). Total scores range from 17 to 85, with interpretations based on both total score and symptom-cluster scores. There are two widely-used versions of the PCL (Weathers et al., 1993; Wilkins et al., 2011): the PCL-M, which measures military-based trauma, and the PCL-C (Conybeare et al., 2012; Karstoft et al., 2014), which measures trauma in civilian settings. The structure of the two questionnaires is virtually identical, with the PCL-M specifically addressing events that occurred during military service. TBI patients, all of whom were veterans, and control subjects with military experience (*N* = 40) were administered the PCL-M. Control subjects without military experience were given the PCL-C (*N* = 132).

The second questionnaire, the CFQ (Broadbent et al., 1982; Bridger et al., 2013), is a 25-item survey that measures the self-reported frequency of cognitive failures on simple tasks of memory, perception, and motor function over the previous 6 months. Subjects are asked to rate the difficulty that they experienced with tasks that are not usually problematic for healthy control subjects (e.g., “Do you find you forget appointments?”) (Reason and Lucas, 1984). Each of the 25 questions is rated on a 5-point Likert scale from 0 (“Never”) to 4 (“Very Often”). Interpretations are based on the total score (0–100) and on separate factor scores (Pollina et al., 1992; Wallace et al., 2002).

**Figure 1:**
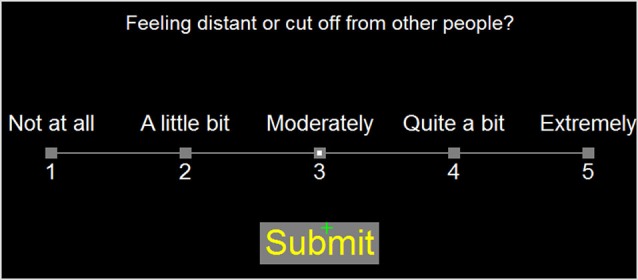
**Question display**. Responses were selected using the mouse cursor (green cross). As the mouse was moved over the rating scale, it highlighted Likert alternatives within a radius of 3.5 degrees of visual angle. Responses remained highlighted when selected by a subject. When satisfied with the response, subjects pressed the submit button. The highlighted Likert rating and “Submit” response are shown.

In the current experiment, the questions from the PCL and CFQ were presented serially by computer. Subjects selected their response to each question with the mouse. Figure 1 shows the computer display. Questionnaire completion was self-paced and subjects were not informed that their completion times would be examined. Subjects read the question and moved the mouse-controlled cursor over the Likert scale to select a rating. When the cursor was near a Likert-scale element, the corresponding response alternative was highlighted. The subject selected the desired rating (indicated by a small white square) by pressing the left mouse button, but could change their rating by selecting another Likert scale value, if desired. Once satisfied with their rating, subjects selected the “Submit” button to proceed to the next question. The time of each button press was recorded. QCTs were measured from the time of question presentation until the final submit response.

Both the PCL and CFQ scales used Likert ratings of 1–5 (rather than the “0–4” scale traditionally used on the CFQ), with rating scores increasing from left to right on both questionnaires (rather than from right to left on the traditional CFQ). CFQ Question 18 was shortened from 36 to 19 words by eliminating an explanatory second sentence, in order to maintain a consistent spatial display structure and font size for all questions. Questionnaire completion took place in a quiet, unsupervised test room and was self-paced.

Testing was performed using a standard PC controlled by Presentation software (Versions 13 and 14, NeuroBehavioral Systems, Berkeley, CA).^2^ Subjects sat 0.7 m from a 17” Samsung Syncmaster LCD monitor, whose refresh rate was 60 Hz. The PCL and CFQ were administered midway through testing with a series of cognitive tests,^3^ with the CFQ delivered 30–35 min after the PCL. Subjects were left alone in the testing room while completing the questionnaire.

### Neuropsychological Evaluation

We selected the results of four other computerized neuropsychological tests for *a priori* comparisons with the QCT results. We chose the WTAR and three tests of executive function: reverse digit span to assess working memory (Woods et al., 2011a), visual CRT to assess processing speed, and the completion time of the trail making test, part B to assess cognitive flexibility (Woods et al., 2015a).

### Statistical Analysis

We corrected for outliers by truncating QCTs greater than 30 s (approximately 0.4% of all trials, maximum 94 s) to 30 s. Nevertheless, the QCT distributions remained highly skewed, so median QCTs were used to characterize subject performance. Because we found that neither PCL scores nor QCTs differed significantly between the groups of control subjects given the PCL-C and PCL-M (*F*_(1,170)_ = 0.92, NS, and *F*_(1,170)_ = 2.35, *p* < 0.15, respectively), the results of PCL-C and PCL-M questionnaires from control subjects were pooled.

The results were analyzed with Pearson correlation and multiple regression. Group comparisons were performed using ANOVA to analyze QCTs and rating scale scores. Greenhouse-Geisser corrections of degrees of freedom were used in computing p values in order to correct for any nonspherical covariation within factors or interactions. Effect sizes are reported as partial *η*^2^ values.

## Results

Table 2 shows median response times averaged across control subjects, showing similar timing on the two questionnaires. On trials where subjects selected a single rating followed by the “submit” response (83% of all trials), subjects required an average of 4.98 s to select the initial rating and 5.97 s to submit their answers. On trials where subjects changed their initial rating with a subsequent choice (12.5% of all trials), the initial selection occurred earlier, at 4.36 s, the rating was changed at 5.64 s, and response submission occurred at 6.51 s. On the remaining trials (approximately 4% of the total), subjects revised their ratings more than once.

**Table 2:** **Response timing**.

	Choice 1	Choice 2	Submit	Percent
PCL	5.07		6.13	83.00%
	4.23	5.63	6.54	12.73%
CFQ	4.89		5.90	83.57%
	4.48	5.64	6.48	12.33%

*Median response times to select a rating and submit responses on trials where only one selection was made (Choice 1), and on trials when the first selection was replaced by a second selection (Choice 2)*.

### The Effects of Question Length and Complexity on Question Completion Time

**Figure 2:**
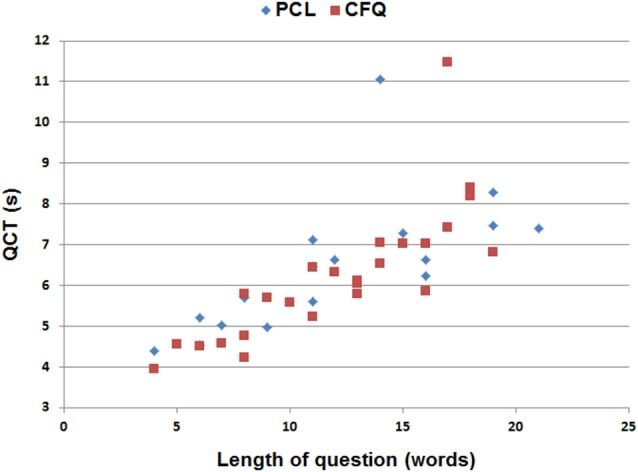
**Median QCTs for the questions on the PCL and cognitive failures questionnaire (CFQ) as a function of the number of words in each question**. The two longest QCTs (top center) occurred to the first question in each questionnaire.

The average question on both the PCL and CFQ questionnaires contained 12.1 words. Figure 2 shows the average median QCTs from the control population for individual questions as a function of question length. QCTs on individual questions correlated strongly with the number of words in the question for both the PCL (*r* = 0.68, *t*_(15)_ = 3.23, *p* < 0.006) and the CFQ (*r* = 0.81, *t*_(23)_ = 6.62, *p* < 0.0001). The first question on both questionnaires (Figure 2, top right) produced longer-latency QCTs than the other questions. When the QCT to the first question was excluded, correlations between QCTs and word counts increased further (PCL: *r* = 0.89, *t*_(14)_ = 7.30, *p* < 0.0001; CFQ: *r* = 0.90, *t*_(22)_ = 9.46, *p* < 0.0001). A closer examination of Figure 2 shows that QCTs increased from approximately 4.5 s in sentences containing five words, to 7.5 s in sentences containing 20 words, implying an average reading rate of approximately five words per second (i.e., 300 words per minute) in the control population as a whole.

In contrast to the contribution of the number of words in the question, reading difficulty had an equivocal effect on QCTs: the Flesch–Kincaid reading grade levels were not significantly correlated with QCTs on the PCL (*r* = −0.01, *t*_(14)_ = −0.04, NS), but they did correlate with QCTs on the CFQ (*r* = 0.53, *t*_(22)_ = 2.92, *p* < 0.01). Thus, question length appeared to be the most important determinant of inter-question differences in QCTs.

### Intersubject Differences in Questionnaire Ratings and QCTs

**Figure 3:**
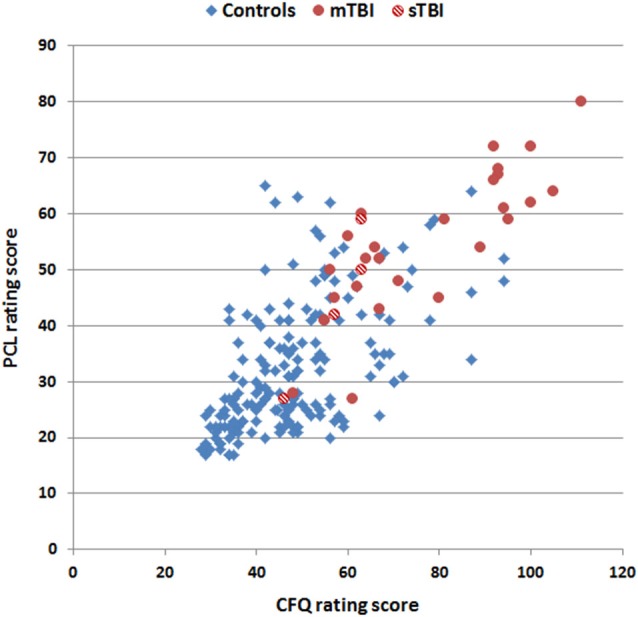
**Rating scores on the CFQ and PCL**. Data are shown for control subjects (blue diamonds), patients with mild TBI (mTBI, filled red circles), and patients with severe TBI (sTBI, cross-hatched red circles).

PCL and CFQ scores, median QCTs, and QCT *z*-scores (see below) from control subjects and TBI patients are included in Table 1. Rating scores on the two tests are shown in Figure 3, and were highly correlated in both control subjects (*r* = 0.56, *t*_(170)_ = 8.94, *p* < 0.0001) and TBI patients (*r* = 0.82, *t*_(29)_ = 7.72, *p* < 0.0001), with TBI patients producing higher disability ratings than controls on both the PCL (*F*_(1,201)_ = 75.92 *p* < 0.0001, partial *η*^2^ = 0.27) and the CFQ (*F*_(1,201)_ = 87.68 *p* < 0.0001, partial *η*^2^ = 0.30).

PCL ratings were weakly correlated with PCL QCTs in control subjects (*r* = 0.20, *t*_(170)_ = 2.66, *p* < 0.01), and no significant correlation was found between CFQ ratings and CFQ QCTs (*r* = 0.04, *t*_(170)_ = 0.52, NS). Likewise, no significant correlations were observed between ratings and QCTs on either test (PCL: *r* = −0.17, *t*_(29)_ = 0.93, NS; CFQ: *r* = −0.31, *t*_(29)_ = −1.76, *p* < 0.10) in the TBI patient group. We also examined within-subject correlations between QCTs and rating scores on individual questions among control subjects and found unsystematic results: some subjects showed positive correlations while others showed negative correlations, resulting in an insignificant average correlation (*r* = 0.03, NS) across subjects. Thus, the magnitude of disability indicated by a subject, either on a question or overall, had minimal influence on QCTs.

### Factors Influencing Question Completion Time

Median QCTs averaged 6.42 s in controls. QCTs varied substantially across subjects and had a positively skewed (2.61) distribution. Therefore, median QCTs were log-transformed to normalize the distribution (resulting skew = 0.90) before further statistical analysis.

**Figure 4:**
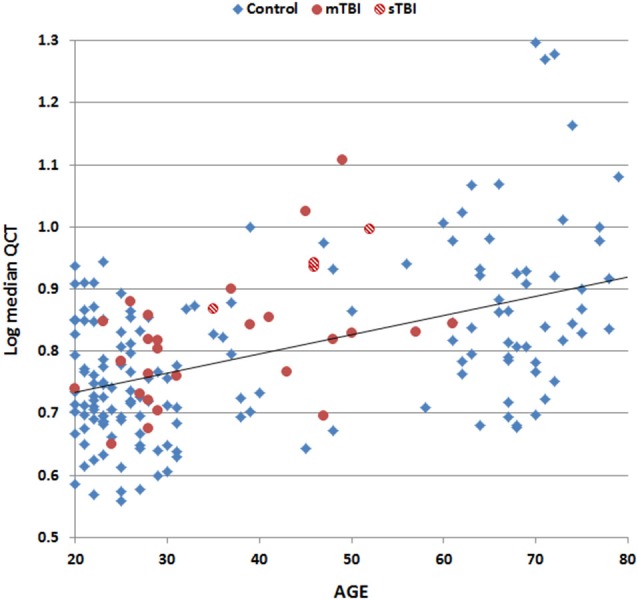
**Log median QCTs (averaged over the PCL and CFQ) as a function of subject age**. Data are shown for control subjects (blue diamonds), patients with mild TBI (mTBI, filled red circles), and patients with severe TBI (sTBI, cross-hatched red circles). The linear fit to control data is shown.

Table 3 shows the correlation matrix for age, education, computer use, rating scores, and log-median QCTs on the two tests. There was a strong correlation between log-median QCTs on the PCL and CFQ (*r* = 0.82, *t*_(170)_ = 18.95, *p* < 0.0001). Figure 4 shows a scatter plot of log-median QCTs (averaged over both questionnaires) as a function of age: log-median QCTs increased with age on both the PCL (*r* = 0.48, *t*_(170)_ = 7.13, *p* < 0.0001) and the CFQ (*r* = 0.47, *t*_(170)_ = 6.94, *p* < 0.0001). As shown in Table 3, QCTs were also reduced with increasing years of education (PCL, *r* = −0.20, *t*_(170)_ = −2.66, *p* < 0.01; CFQ, *r* = −0.16, *t*_(170)_ = −2.11, *p* < 0.05). Log-median QCTs were shorter in female subjects on both tests (PCL: *F*_(1,170)_ = 8.60, *p* < 0.004, *η*^2^ = 0.05; CFQ: *F*_(1,170)_ = 16.62, *p* < 0.0001, *η*^2^ = 0.09).

**Table 3:** **Correlation matrix for control subjects**.

	Edu	Sex	C-use	PCL	CFQ	PCL LQCT	CFQ LQCT
Age	0.17	0.14	−0.18	−0.13	−0.18	0.48	0.47
Edu		0.01	0.30	−0.29	−0.16	−0.20	−0.16
Sex			−0.04	0.12	0.24	0.22	0.30
C-use				−0.19	−0.08	−0.53	−0.48
PCL					0.56	0.24	0.08
CFQ						0.12	0.08
PCL LQCT							0.82

*Education (EDU), sex (female = 0, male = 1), computer use (C-use), rating scores on the PCL and CFQ, and log-median QCTs (LQCTs) on the PCL and CFQ. Given the size of the control subject population (*n* = 172), |r| = 0.15 is significant at *p* < 0.05 and |r| = 0.30 is significant at *p* < 0.0001*.

Computer use, which correlated positively with education (*r* = 0.30, *t*_(170)_ = 4.10, *p* < 0.0001) and negatively with age (*r* = −0.18, *t*_(170)_ = −2.34, *p* < 0.02), also had a large effect on QCTs (PCL: *r* = −0.53, *t*_(170)_ = −7.94, *p* < 0.0001; CFQ: *r* = −0.48, *t*_(170)_ = −6.76, *p* < 0.0001). Finally, log-median QCTs on the PCL were slightly increased in subjects with higher mean PCL ratings (*r* = 0.24, *t*_(170)_ = 3.22, *p* < 0.002), but rating scores on the CFQ did not correlate significantly with log-median CFQ QCTs (*r* = 0.08, *t*_(170)_ = 1.05, NS).

Multiple regression was used to analyze the combined effects of age, education, sex, and computer use on log-median QCTs. On the PCL, these four factors conjointly accounted for 44.0% of the log-median QCT variance (*r* = 0.66, *F*_(4,167)_ = 32.84, *p* < 0.0001), with large effects of age (*t*_(167)_ = 5.91 *p* < 0.0001) and computer use (*t*_(167)_ = −5.78, *p* < 0.0001), and smaller, but still significant effects of education (*t*_(167)_ = −2.35, *p* < 0.02) and sex (*t*_(167)_ = 2.63, *p* < 0.01). On the CFQ, these four factors accounted for 41.7% of the log-median QCT variance (*r* = 0.65, *F*_(4,167)_ = 29.9, *p* < 0.0001), with age (*t*_(167)_ = 5.68, *p* < 0.0001) and computer use (*t*_(167)_ = −4.93, *p* < 0.0001) again having larger influences than education (*t*_(167)_ = −1.93, *p* < 0.06) or sex (*t*_(167)_ = 3.92, *p* < 0.0002).

**Figure 5:**
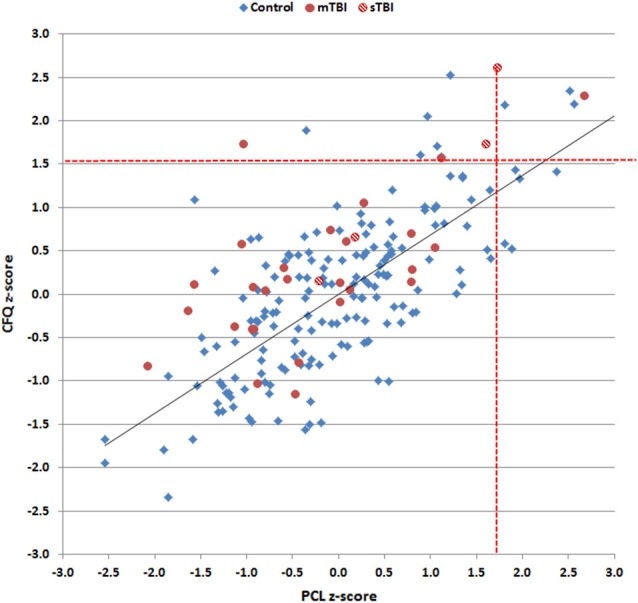
**Log-median question completion time (QCT) *z*-scores for control subjects and TBI patients on the PCL and CFQ**. Linear regression was used to correct for the influence of age, education, computer use, and sex on *z*-scores. Red dashed lines show the upper limits (*p* < 0.05) of the control population. Data are shown for control subjects (blue diamonds), patients with mild TBI (mTBI, filled red circles), and patients with severe TBI (sTBI, cross-hatched red circles). The linear fit to control data is shown.

Figure 5 shows *z*-score measures of log-median QCT *z*-scores in control subjects and TBI patients after regressing out the effects of age, education, sex, and computer use. CFQ and PCL QCT *z*-scores showed strong correlations with each other in controls (*r* = 0.70, *t*_(170)_ = 12.78, *p* < 0.0001) and TBI patients (*r* = 0.81, *t*_(29)_ = 7.44, *p* < 0.0001).

### Correlations of QCTs with the Results of other Neuropsychological Tests

Table 4 shows the correlations between log-median QCTs and QCT *z*-scores and the results of other cognitive tests. Log-median QCTs correlated significantly with all other tests, including the WTAR (PCL: *r* = −0.42, *t*_(168)_^4^ = −6.03, *p* < 0.0001; CFQ: *r* = −0.41, *t*_(168)_ = −5, 86, *p* < 0.0001), reverse digit span (PCL: *r* = −0.35, *t*_(168)_ = −4.84, *p* < 0.0001; CFQ: *r* = −0.27, *t*_(168)_ = −3.64, *p* < 0.0004), choice reaction time (PCL: *r* = 0.34, *t*_(147)_ = 4.71, *p* < 0.0001; CFQ: *r* = 0.30, *t*_(147)_ = 4.10, *p* < 0.0001), and particularly Trails B (PCL: *r* = 0.67, *t*_(164)_ = 11.77; CFQ: *r* = 0.65, *t*_(164)_ = 11.15, *p* < 0.0001 for both comparisons). However, the correlations between the log-median QCTs on the two questionnaires (*r* = 0.82) significantly exceeded the correlations between log-median QCTs and the results of any other cognitive test (i.e., *z* = 3.18, *p* < 0.002 for comparisons with the *r* = 0.67 correlation observed for Trails B).

**Table 4:** **Correlations of QCTs with other neuropsychological tests**.

	CRT	WTAR	DSR	Trails B
PCL LQCT	0.34	−0.42	−0.35	0.67
CFQ LQCT	0.30	−0.41	−0.27	0.65
PCL QCT-z	0.27	−0.34	−0.20	0.40
CFQ QCT-z	0.24	−0.33	−0.10	0.38

*Correlations of log QCTs and QCT *z*-scores on the PCL and CFQ with choice reaction time (CRT), the Wechsler Test of Adult Reading (WTAR), reverse digit span (DSR), and the trail making test part B (Trails B)*.

Corrections of the QCTs for age, education, sex, and computer use reduced the correlations with other cognitive measures (Table 4), which nevertheless remained significant for choice reaction time, the WTAR, and particularly Trails B (e.g., *r* = 0.40 with QCT *z*-scores on the PCL). Again, the correlation between the QCT *z*-scores on the two questionnaires (*r* = 0.70) significantly exceeded the correlations seen with any other cognitive test (*z* = 4.08, *p* < 0.0001).

### QCT Analysis of TBI Patients

A comparison of QCT *z*-scores between control subjects and the TBI patient group as a whole showed no significant group differences on either the PCL (*F*_(1,201)_ = 0.53, NS) or the CFQ (*F*_(1,201)_ = 2.99, *p* < 0.09). However, the differences between CFQ and PCL *z*-scores were significantly larger in the TBI patient group as a whole than in controls (*F*_(1,201)_ = 9.33, *p* < 0.003, partial *η*^2^ = 0.04): i.e., patients showed relatively increased QCTs on the CFQ compared to the PCL.

Further comparisons of TBI patient subgroups showed no significant differences in QCTs between control subjects and mTBI patients on either test (PCL, *F*_(1,197)_ = 1.69, NS; CFQ, *F*_(1,197)_ = 1.12, NS). However, comparisons of sTBI patients and controls showed a trend toward elevated QCTs on the PCL (*F*_(1,74)_ = 2.05, *p* < 0.16) and a significant increase in QCTs on the CFQ (*F*_(1,74)_ = 4.95, *p* < 0.03, *η*^2^ = 0.03). Comparisons of mTBI and sTBI patients showed a borderline QCT increase in sTBI patients on both the PCL (*F*_(1,29)_ = 4.33, *p* < 0.08) and the CFQ (*F*_(1,29)_ = 4.14, *p* < 0.06, *η*^2^ = 0.12).

## Discussion

Questionnaires are commonly used to gather demographic and clinical data. Here, we demonstrate that QCTs also provide a useful metric of cognitive and executive function that reflects reading speed, speed of decision making, and speed of motor execution. Importantly, QCT measures provide a metric of processing speed and executive function in a self-paced task, and hence also reflect motivation and effort as well as cognitive ability.

Unsurprisingly, QCTs correlated significantly with the results of other cognitive tests. However, stronger correlations were seen between the QCTs on the two questionnaires than between either questionnaire and other cognitive measures. This suggests that QCTs provide insights into cognitive and executive functions that are not fully captured by other common neuropsychological tests (Reitan and Wolfson, 2005).

### Stages of Question Completion

Completing each question required that subjects read the question, decide on an appropriate rating, choose an appropriate response, select the chosen rating with the mouse, alter the decision if necessary by selecting a different response, and finally submit the rating before moving on to the next question. QCTs on the shortest (4–6 word) questions averaged approximately 4.5 s. Since average reading speed was approximately five words/s, a five-word question would require about 1.0 s to read, and approximately 1.0 s was needed to move the cursor and execute the submit response. Assuming that the selection of the initial rating also required 1.0 s, subjects would appear to take approximately 1.5 s to decide on an appropriate rating. Of course, the relative timing of different processing stages would vary with question length. For example, on sentences of average length (12.1 words), reading would require approximately 2.4 s, or more than one-third of overall completion time (6.5 s).

We found that QCTs to individual questions correlated strongly with the number of words in each question, indicating that a substantial portion of QCT variance reflected the time needed to read each question. QCTs also varied with subjects’ age, which is known to influence overall questionnaire completion times (Yarnold et al., 1996; Malhotra, 2008) and reading speed (Tiu et al., 2003; Borella et al., 2011; Caplan et al., 2011). Age also influences the speed with which subjects move the mouse cursor in the Trail Making Test (Woods et al., 2015a), whose completion times were strongly correlated with QCTs.

QCTs showed significant correlations with education, which has well-established influences on performance in cognitive tasks (Schneider et al., 2015). In addition, QCTs were faster in women than men, consistent with a large body of evidence indicating superior female reading ability (Lynn and Mikk, 2009). Finally, we found strong correlations between QCTs and computer use. Presumably, experienced computer users were both more familiar with manipulating the mouse and more experienced in completing computerized questionnaires. In addition, computer use correlates strongly with performance on non-computerized tests such as verbal fluency, digit span, and measures of crystallized intelligence such as the WTAR. Multiple regression analysis demonstrated that age, computer use, sex, and education all had significant influences on QCT performance, with computer use exerting nearly as strong an influence as age.

Control subjects who produced higher mean questionnaire ratings, indicating higher levels of subjective cognitive impairment, showed a slight increase in QCT *z*-scores on the PCL, but not the CFQ, and no significant correlations were found between ratings and QCTs among TBI patients. Moreover, the QCTs of individual subjects did not correlate with disability ratings. This suggests that QCTs provide information about the speed and efficiency of questionnaire completion that is largely independent of subjective ratings.

Recent reports have called attention to the importance of developing novel instruments to assess executive function in ecologically valid settings (Lamberts et al., 2010; Novakovic-Agopian et al., 2014). QCTs directly measure performance in the self-paced task of completing computerized questionnaires, and can be obtained incidentally during questionnaire administration. Moreover, since questionnaire completion is self-paced, QCTs provide insight into the utilization of cognitive resources in relatively unstructured settings (Reitan and Wolfson, 2000) that may reflect energetical aspects of executive function (Stuss, 2011).

We found slowed QCTs of sTBI patients on the CFQ, and borderline slowing on the PCL questionnaire. We also observed larger differences between CFQ and PCL QCTs in the TBI patient group as a whole when compared to control subjects. One possible explanation for these discrepancies is that the TBI patients had been repeatedly exposed to paper-and-pencil versions of the PCL during their clinical evaluation and treatment; i.e., QCTs in TBI patients may have been reduced on the PCL due to their familiarity with the PCL questions. No such reduction would have occurred for the unfamiliar questions on the CFQ, explaining the difference in QCTs between the two tests.

### Limitations

The correlations between QCTs on different questionnaires, as well as the correlations between QCTs and the results of other cognitive tests, are likely to vary with questionnaire structure and content. For example, questionnaires varying in the average number of words in each question would alter the relative contribution of reading speed to overall QCT. In addition, the distinctiveness of different response foils might change cognitive demands and alter speed/accuracy tradeoffs. For example, when asked “How much wine to you consume daily?” an individual might more rapidly choose “one glass” in comparison with “0.12 liters”. Similarly, multiple choice and “select all that apply” questions would be expected to introduce different cognitive demands than those associated with Likert scale judgments, thus adding scoring complexity due to different numbers of responses from different subjects. Finally, further studies using larger clinical populations with TBI and other neurological and psychiatric disorders are needed to more fully evaluate the utility of QCT measures of cognitive function in clinical populations.

## Conclusions

Question Completion Time provides a useful and reliable measure of cognitive and executive function that can be gathered in parallel with demographic and clinical information when questionnaires are administered. QCTs reflect reading ability, processing speed, executive control, and the ability of subjects to focus resources in a self-paced task. QCTs are largely determined by reading speed, as seen in the strong correlation between the QCTs and the number of words in individual questions. Strong correlations were also found between QCTs on two different questionnaires, which significantly exceeded the correlations of QCTs on either questionnaire with the results of other cognitive tests. This suggests that QCTs provide insight into cognitive and executive functions that complement the results of other neuropsychological instruments. Our results suggest that QCTs are prolonged in patients who have suffered severe TBI. However, additional studies with larger populations are needed to further evaluate the clinical sensitivity of QCTs to traumatic brain injury and other causes of cognitive impairments, and to determine the extent to which QCTs predict performance in other cognitively-demanding, self-paced tasks.

## Conflict of Interest Statement

Dr. Woods is affiliated with NeuroBehavioral Systems, Inc., the developers of Presentation software used to program these experiments.

## Footnotes

^1^sfbay.craigslist.org

^2^The computerized versions of the PCL and CFQ questionnaires are available at www.ebire.org/hcnlab/

^3^These included the following computerized tests and questionnaires: finger tapping, simple reaction time, Stroop, digit span forward and backward, phonemic and semantic verbal fluency, verbal list learning, spatial span, trail making, design fluency, the Wechsler Test of Adult Reading (WTAR), visual feature conjunction, the Paced Auditory Serial Addition Task (PASAT), and a traumatic brain injury (TBI) questionnaire.

^4^Data were not available on all tests from subjects who completed the questionnaires.
